# Reproducibility of grading systems in oral epithelial dysplasia

**DOI:** 10.4317/medoral.17749

**Published:** 2012-05-01

**Authors:** Adesh Manchanda, Devi-Charan Shetty

**Affiliations:** 1 BDS, MDS, Senior Lecturer, Department of Oral and Maxillofacial Pathology and Microbiology, Sri Guru Ram Das Institute of Dental Sciences and Research, Amritsar, India; 2 BDS, MDS, Professor, Department of Oral and Maxillofacial Pathology and Microbiology, I.T.S Center for Dental Studies and Research, Muradnagar, Ghaziabad, India; 3 ….

## Abstract

Objective: To assess inter and intra observer variability in grading oral epithelial dysplasia (OED) using Smith and Pindborg grading system, WHO classification system and Brothwell DJ et al. classification system. Study design: In the study 45 histological tissues of dysplasia, 15 each of mild, moderate and severe dysplasia were coded and blindly graded by three observers in three grading systems. Further on the same observers graded 15 slides again of the previous 45 for analyzing the reproducibility in the three grading systems. The individual significance of various indicators of dysplasia among various grades of dysplasia was also assessed. Result: Inter observer agreement was significantly higher in Brothwell system as compared to WHO and Smith and Pindborg system. Intra observer agreement was significantly higher in Smith and Pindborg system, but the predictability and the probability index was distributed over a larger range in this system. Each indicator of dysplasia was also found to be statistically significant (P<0.05) for grading dysplasia. Conclusion: The present study puts forth the inherent intricacies in the grading of oral premalignant lesions.

** Key words:**Carcinoma, dysplasia, grading systems, reproducibility.

## Introduction

The term cancer, by itself has evoked a sense of morbidity and mortality among the medical fraternity as well as in general population. Cancer of the mouth is a serious condition; with just over half of the afflicted individuals surviving for over 5 years (1). In the oral cavity the appearance of cancer is preceded by other lesions which may show various tissue morphological changes and histopathological cellular changes that point towards the possible subsequent development of malignancy. The most important of these recognizable changes are dysplastic changes which would merit this altered tissue in the oral cavity to be stated as a premalignant lesion (1).

Potentially malignant disorders, namely leukoplakia and erythroplakia have a proportion of becoming overtly malignant; this subgroup might then reasonably be termed as ‘premalignant lesion’, whereas the remainder may not become malignant within the life span of patient. Leukoplakia is a predominantly white lesion of the oral mucosa that cannot be characterized as any other definable lesion (2). The cellular mainstay of this potentially malignant disorder is the presence of dysplasia.

Oral epithelial dysplasia (OED) thus is the most important recognizable change microscopically from a normal epithelium to a diseased entity. The lesions are graded into different categories and this grading process is basically structured on the potential risk of malignant transformation (3). Follow up studies have shown varying malignant transformation rates ranging from 4.4% to 17.5% (4).

In grading of OED a perspective of the whole tissue in general is reached (i.e. both cell to cell and layer to layer changes of the epithelium which is of advantage over exfoliative cytology). Many systems of grading epithelial dysplasia have been proposed in order to standardize the severity of dysplastic features. Any grading system would be clinically useful if they are reproducible between separate observers. Smith and Pindborg (5) pointed out that the recognition and interpretation of the features of epithelial dysplasia and the assessment of their degree of importance vary not only from observer to observer, but also in the same observer from day to day (5).

Substantial variation has been reported in the grading of oral epithelial dysplasia and standardization is one of the greatest prob-lems in assessing epithelial dysplasia, as is establishing the relative importance of different clinical and dysplastic features (5). The accuracy and precision of a measurement or diagnosis is normally evaluated by looking at the validity and reproducibility of examiner observations, respectively (1).

When studying the accuracy obtained in grading oral epithelial dysplasia, there is no test available, which is thought to be better than pathologist’s observation, an accepted gold standard is not available for assessing the validity obtained when grading OED (1). Although one study has shown the high predictive value of DNA aneuploidy in OED, histopathological evaluation based on morphology remains the routine method for diagnosis and grading (6).

Interestingly, the subjectivity in the evaluation of the established criteria of grading, arbitrary division of the grading, lack of calibration of the used criteria and grading, and the lack of sufficient knowledge of which criteria are important for the prediction of malignant potential are attributed for the lack of agreement on grading oral dysplasia lesions (7,8).

Grading, in fact, is an attempt to impose discrete ca-tegories on what is in effect a continuous grey scale. Any grading effort is therefore by definition artificial. Pathologists need to provide information which is useful to clinicians, but are asked to do so by artificially creating discrete sub-entities in a biological continuum. Unless clear criteria are decided upon, which will always be artificial, this act will remain poorly reproducible, not because of the incapacity of pathologists, but because of the nature of the biological process (9).

A working model in grading dysplasia shows, that reducing the number of categories leads to an increase in inter observer agreement, as measured by kappa statistics, but a decrease in information transmitted (10).

Thus, the present study was initiated to dwell on the subject of reluctance seen by the observer himself to his own assessment of dysplastic lesions. The study approached this problem of deciphering clarity by applying inter observer assessments and their analysis. Another concern of the study was to highlight the grey areas using quantifiable yardsticks which need to be standardized in varying grading systems such that a uniform assessment is achievable when these differing systems were used.

We aimed to assess inter and intra observer variability in grading oral epithelial dysplasia using Smith and Pindborg grading system (5), WHO classification system (8) and Brothwell DJ et al. classification system (1). Along with this the individual significance of various indicators of dysplasia with respect to their reliability among various grades of dysplasia was categorized.

## Material and Methods

-Case selection

Forty five histologic sections of OED were selected from departmental archives by a non participating Oral Pathologist in the department of Oral Pathology, ITS-CDSR. The tissues given were previously reported for mild, moderate and severe dysplasia (sign out diagnosis). The sign out diagnosis was given by a collaborative agreement of all the examiners when the slide was viewed collectively.

-Inclusion Criteria

To be selected for the current study, sections had to meet the following criteria: acceptable diagnostic quality, intraoral site and included referral information on age, sex and site of the lesion as clinical details for a sign out diagnosis. The final 45 cases were originally signed out as follows: 15 with mild dysplasia, 15 with moderate dysplasia and 15 with severe dysplasia. All the cases were coded and randomly arranged for all the examiners and examinations. The pathologists were not provided with any demographical information regarding the expected distribution of severity of the cases.

-Examiner/Examinations

Three certified oral pathologists all belonging to the department of Oral and Maxillofacial Pathology and Microbiology of I.T.S Centre for Dental Studies and Research, Muradnagar, Ghaziabad (U.P) affiliated to Chaudhary Charan Singh University, Meerut assessed and graded the 45 cases. For each case, a single 4 µm thick tissue section stained with Harris haematoxylin and eosin was examined by light microcopy. No special stains or deeper sections were made available. Referral information on age, sex, smoking habit, tobacco chewing status or amount of alcohol consumption and anatomical site of the lesion for each case were not provided. The examination of the cases was carried out based on three grading systems of dysplasia and the observations in each of the grading systems were compared at the end. The individu-al significance of various indicators of dysplasia among various grades of dysplasia was also assessed.

-Study Design

In the first phase of the study, the observers were given all 45 cases individually without the sign out diagnosis with slides marked randomly with a coded labeling system, the details of which were accessible only to the non participating oral pathologist. Observers were asked to complete the examination within a 2 month period and to assess each slide for the presence and severity of dysplasia based on the following systems: Smith and Pindborg system, WHO system, Brothwell D J et al. system.

Smith and Pindborg System (5) attempt to standardize the grading of dysplasia by photographically obser-ving each microscopic feature at a time and allocating a weighted score to each feature. Each feature is graded as absent, slight and marked.

The features of dysplasia along with its severity accor-ding to Smith and Pindborg System are tabulated ( Table 1).

**Table 1:**
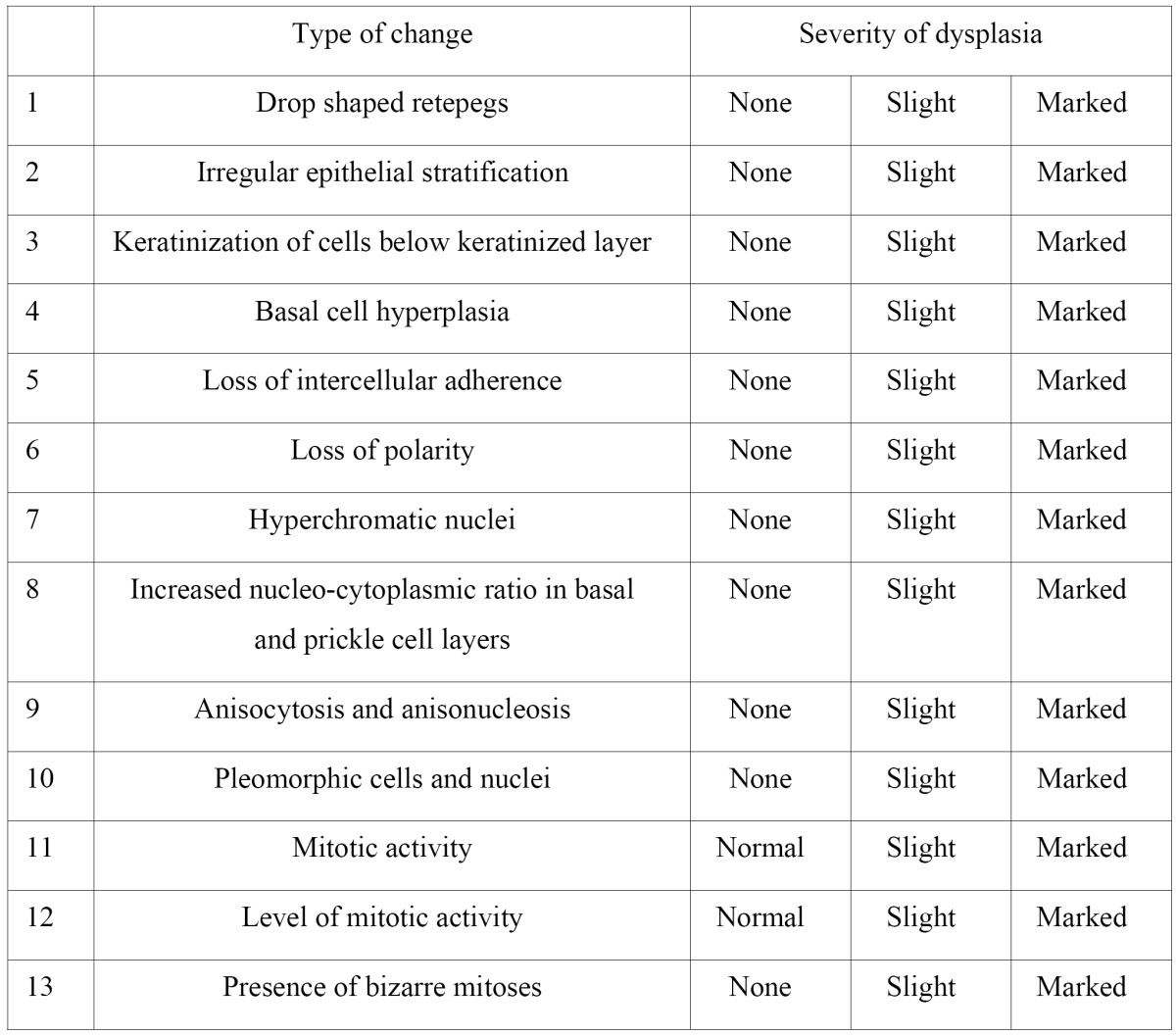
The features of dysplasia according to Smith and Pindborg System.

-Scoring

Grading of ‘none’ is scored – 0

Grading of ‘slight’ or ‘marked’ is scored – 1 to 10

Total score of all features is taken as Epithelial Dysplasia Index (EDI). It can vary from 0 – 75.

The grading finally is done as follows: (Table  Table 2)

**Table 2:**
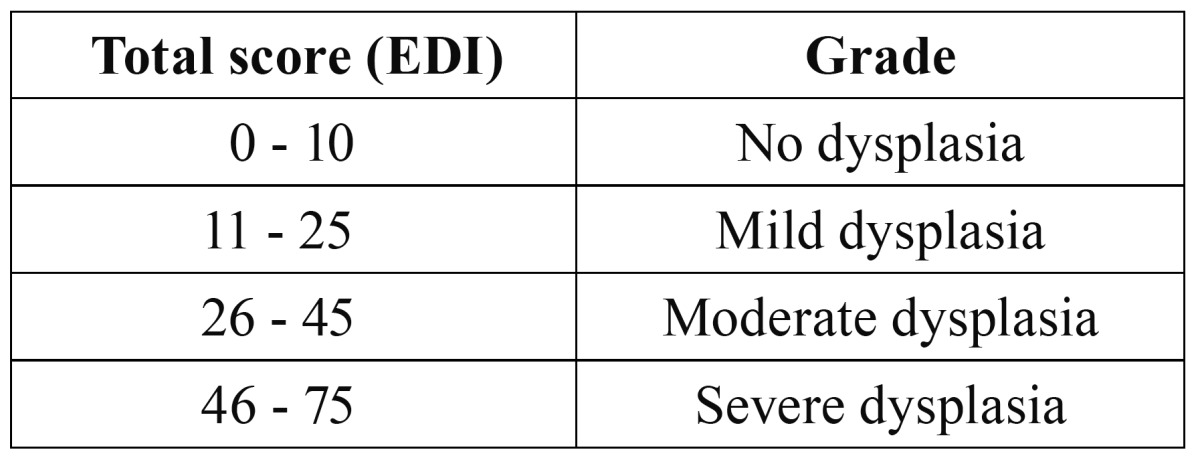
Scoring of dysplasia.

The standardization of Smith and Pindborg System was done collaboratively by the oral pathologists of the department.

WHO System (8) defines and lists out 12 histologic characteristics that characterize the epithelial dysplasia into grades of mild, moderate and severe.

Mild dysplasia: slight nuclear abnormalities, most marked in the basal third of the epithelial thickness and minimal in the upper layers, where the cells show maturation and stratification. A few, but no abnormal mitoses may be present, usually accompanied by keratosis and chronic inflammation.

Moderate dysplasia: More marked nuclear abnormalities and nucleoli tend to be present, with changes most marked in the basal 2/3rd of the epithelium, nuclear abnormalities may persist up to the surface, but cell maturation and stratification are evident in the upper layers. Mitoses are present in the parabasal and intermediate layers, but none is abnormal.

Severe dysplasia: Marked nuclear abnormalities and loss of maturation involving more than 2/3rd of the epithelium, with some stratification of the most superficial layers. Mitoses some of which are abnormal may be present in the upper layers.

Brothwell D J et al. System (1): A 5 point scale grading system was used for oral epithelial dysplasia. The grading scale criteria are as follows:

0= No dysplasia

1= Mild dysplasia: Increased number of cells in the basal and parabasal epithelial regions showing nuclear hyperchromatism and pleomorphism.

2= Moderate dysplasia: Bulbous retepegs with increased number of cells showing hyperchromatism and pleomorphism, extending to and including the basal, parabasal and prickle cell layer.

3= Severe dysplasia: Bulbous retepegs with increased number of cells showing nuclear hyperchromatism and pleomorphism through the entire thickness of epithelium.

4= Carcinoma in situ: Markedly atypical changes showing nuclear hyperchromatism and pleomorphism and encompassing the entire thickness of the epithelium, with the suggestion of early superficial connective tissue invasion, but without convincing evidence.

After grading according to the 3 systems, the data was collected from the individual observers and compared for interobserver variability. To assess the intra observer agreement the same examiners were again given 15 slides of the previous 45 cases. Computations were done to analyze the reproducibility of these individually observed diagnoses.

-Statistical analysis

The data collected was first visualized to confirm their normal distribution. The resulting data was analyzed using SPSS version 10 and Epi-Info 6.04 d software. Following this, descriptive statistics including the mean values and standard deviations, 95% confidence intervals, interquartile ranges (25th and 75th percentiles) were calculated for each variable. Pearson chi square test was carried out to determine the level of correlation or association between the groups under study. The unweighted kappa statistics was applied to observe the agreement between the observers. Differences between the different variables were analyzed using Anova test and Post Hoc test. Beside this Kruskal-Wallis one way test was also applied to compare skewed data among the groups followed by Mann-Whitney U test adjusted for probabilities. P value <0.05 was considered as significant.

To interpret the quantitative significance of kappa (11) the following guidelines were used: Cohen’s unweighted Kappa statistic (Ks) <0, poor agreement; Ks 0.0-0.20, slight agreement; Ks 0.21-0.40, fair agreement; Ks 0.41-0.60, moderate agreement; Ks 0.61-0.80, substantial agreement; Ks 0.81-1.00; almost perfect.

-Ethical consideration

A full description of the study protocol was submitted to the ethical review committee, Chaudhary Charan Singh University. Written notification was received from the University, confirming that, as a result of the negligible risk for involved patients, no formal ethical approval was necessary.

## Results

In common with all other studies assessing observer variability, examiner agreement levels demonstrated in this study were less than perfect.

-Extent of observer agreement

Interobserver agreement in Smith and Pindborg system, WHO system and Brothwell system

In order to compare the agreement among individual pathologists in each of the three systems the unweighted kappa statistics was calculated which was found to be significant in all three systems (p<0.05). A good interobserver agreement was seen in Brothwell system (P= 0.0226) which had the highest kappa value (1018). Agreement (P= 0.0346) in WHO system showed a kappa value of .1156 while least agreement (P= 0.0486) between the three observers was seen in Smith and Pindborg system with a kappa value of 0.0920.

-Intraobserver agreement in Smith and Pindborg system, WHO system and Brothwell system

The interobserver agreement showed a larger range of va-lues for agreement (60.0% -93.3%) ( Table 3). The accuracy of reproducing the observations though high in individual observers, the predictability as measured through the kappa values and the probability index were distributed in a larger range. The WHO system also showed a significant intraobserver agreement ( Table 3). Although the interobserver agreement between the three observers was best seen in Brothwell system, the intra observer agreement was less significant with an agreement range from 53.33% to 66.67% ( Table 3). The least variability in range between the three observers was seen in this system.

**Table 3:**
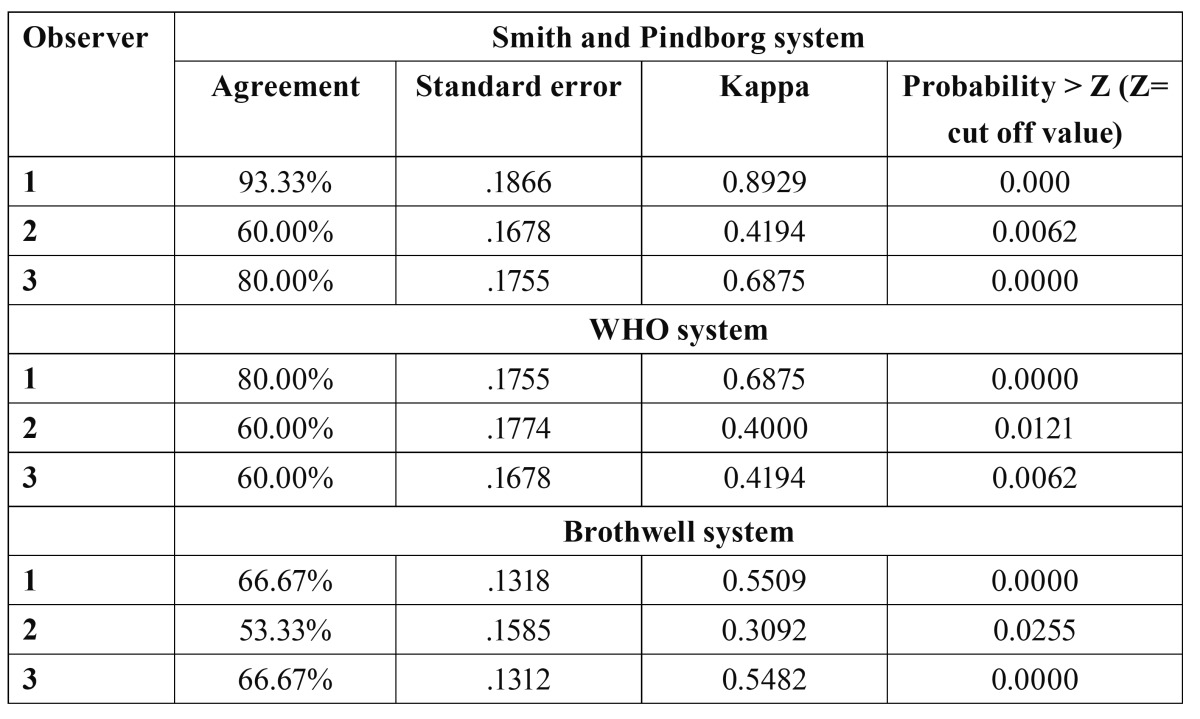
Intraobserver agreement of the three observers in Smith and Pindborg system, WHO system and Brothwell system.

-Individual significance of various indicators of dysplasia in grading

The significance of each indicator of dysplasia was calculated in differentiating mild dysplasia from moderate dysplasia, mild dysplasia from severe dysplasia and mode-rate dysplasia from severe dysplasia ( Table 4). Indicators of dysplasia were found significant in differentiating between the various grades of dysplasia except drop shaped retepegs, basal cell hyperplasia, loss of intercellular adherence and loss of polarity which were not significant in differentiating moderate from severe dysplasia with a P value = 0.935, 1.000, 0.239 and 0.0999 respectively. Presence of bizarre mitosis was not significant in differentiating mild from moderate dysplasia (P=0.183).

**Table 4:**
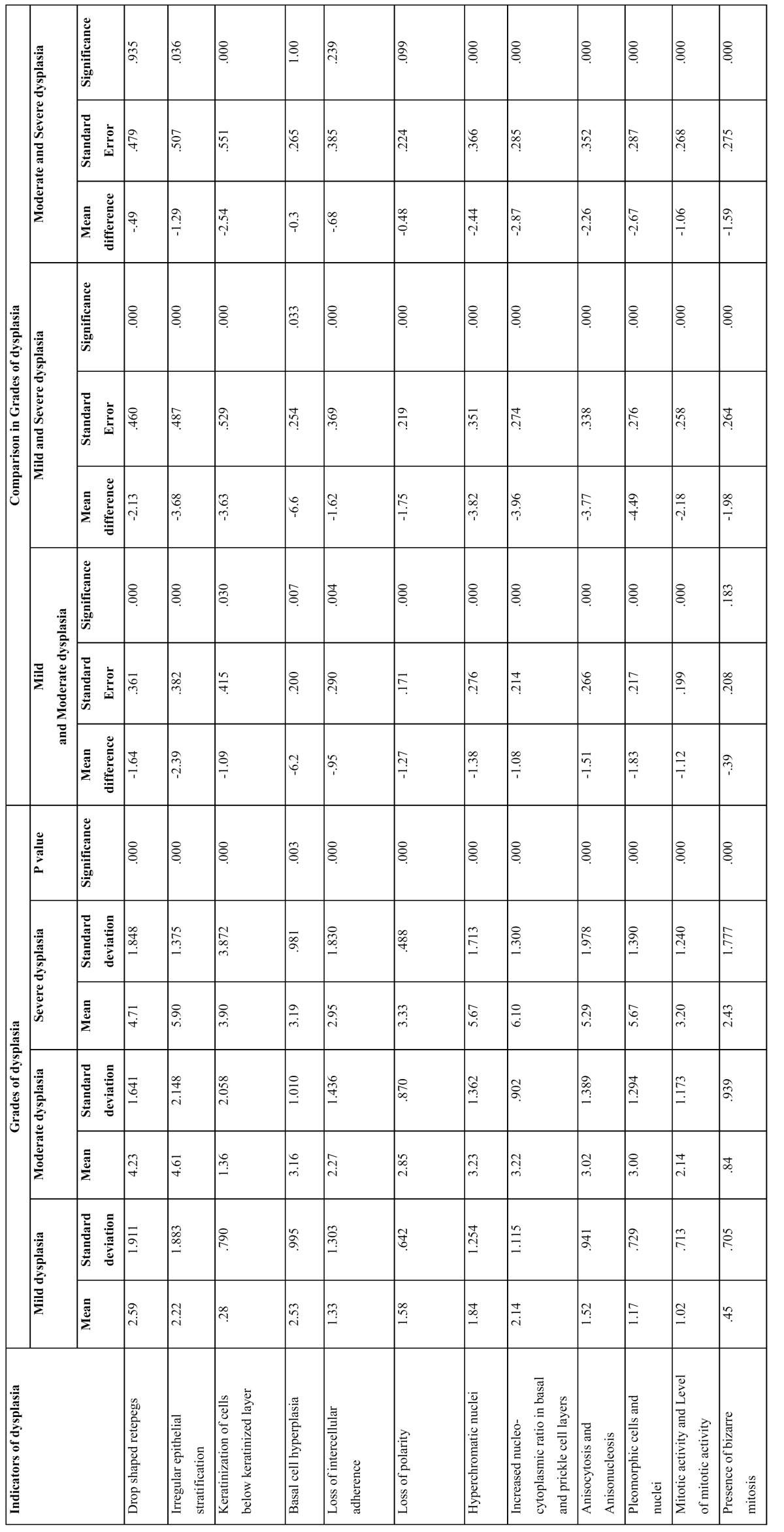
Individual significance of various indicators of dysplasia in different grades and its reliability among various grades of dysplasia.

## Discussion

Oral carcinomas frequently arise from a spectrum of abnormalities ranging from hyperplasia to intraepithelial neoplasia termed histopathologically oral epithelial dysplasia (OED) (3). In head and neck pathology, the term dysplasia is increasingly used. In standard medical terminology, dysplasia means an abnormality of development, while in histomorphology it expresses cellular and structural changes of the epithelium. Considering these abnormalities as typical of the progression from normal epithelium to cancer, the lesions are graded into different risk groups (12).

Grading of dysplasia is demanded almost daily from most diagnostically active pathologists. It is also notoriously subjective and lacks inter and intraobserver reproducibility. This is partly due to the lack of validated morphological criteria, upon which pathologists have reached consensus. It is largely due to the biological nature of the evolution of dysplasia, not in discrete steps, but as a continuum (9).

The clinical observation of the malignant transformation in a certain group of oral lesions has led to the concept of defining these lesions as premalignant oral lesions. Interestingly, over time, there was inconsisten-cy in using uniform terms globally to describe these lesions. In fact, several terms have been used, currently; the 2005 WHO classification terms these as precursor oral lesions. Histopathologically, the architectural and cytological features that are found in cancer have been used as indicative of “premalignancy” or “precursor” lesions. In similar fashion to clinicians, pathologists have a wide variety of terms to describe these changes; the most common of these were “dyskeratosis”, “atypia” and “dysplasia”. This confusion in terminology has lead to inconsistency in the reporting of oral epithelial dysplasia lesions by pathologists. Moreover, since the introduction of defined architectural and cytological criteria, there has hardly been a study aimed at evaluation of how significant these changes are in diagnosing and grading oral epithelial dysplasia (3).

In the diagnosis of oral epithelial dysplasia it is customary to distinguish between various grades. However, the histopathologic diagnosis is often biased by incorporation of the clinical facts and the description from the clinician. The clinician often uses terms such as “histologic verification” of the clinical diagnosis and the histopathologists diagnosis and grading of dysplasia are often used as a “gold standard”. When the clinician interprets the histopathologic diagnosis, he or she should be aware that the histopathologist may be influenced by the clinical findings and thereby avoid a double weighting (13).

Several studies have shown great variability in inter and intra-observer agreement in the diagnosis and grading of oral epithelial dysplasia. Their results ranged from poor to substantial agreement using different statistical methods. Pindborg et al. (14) first reported on the observer agreement in the assessment of OED lesions. Nine photomicrographs were evaluated by 72 pathologists at a scientific meeting of oral pathologists. A wide range of agreement was reported (1-78%). The results of this study could not be generalized due to lack of statistical analysis and the participation of observers that were not experienced oral pathologists (15).

The results of a study involving four pathologists, each examining 100 consecutive cases of oral leukoplakia, were reported in 1995. Interobserver percentage agreement ranged from 49 to 69% between the different pathologist pairs with Ks values ranging from 0.27 to 0.45, showing poor to moderate agreement at best. Unfortunately in the study intraobserver agreement was not reported (13).

Brothwell et al. (1) showed a substantial agreement using a 5 point ordinal scale with a group weighted kappa (Kw) of 0.74 (95% Confidence Interval (CI) = 0.64 - 0.85). However similar to other studies, the group unweighted kappa showed a fair to moderate agreement (Ks) of 0.37 with 95% CI = 0.32 – 0.42). Likewise, Fischer et al. (16) reported an overall kappa weighted value of 0.59 (95% CI= 0.45 - 0.72) and an improvement in the kappa agreement was observed when the histological diagnosis was simplified into three general categories of no abnorma-lity/hyperplasia, mild/moderate/severe dysplasia and carcinoma in situ Kw = 0.70 (95% CI= 0.56 – 0.84). However similar to other studies, subjective judgment and individual histopathological experience of the participating pathologists was the core for the histopathological assessment of the studied slides (15).

The data presented in these studies demonstrate the subjectivity among pathologists in the diagnosis of OED. Greater objectivity and standardization in diagnosing epithelial dysplasia are certainly needed. Others have tried unsuccessfully to use standardized photographs, genetics, cytometry, and computers to introduce objectivity into the analysis of epithelial dysplasia. Perhaps the nature of epithelial dysplasia itself and the scantiness of knowledge about the transformation of cells through a pre-neoplastic phase to frank neoplasia are the factors that are frustrating pathologists. The tissues in epithelial dysplasia are in transition, going through a state of morphologic ambiguity in which the exact mechanism that directs a cell toward either normalcy or neoplasia is unknown (17).

In a molecular study by Proliferating cell nuclear antigen (PCNA) on potentially malignant disorders and squamous cell carcinoma, it was confirmed that higher the cell proliferation rate, the higher the risk of cells suffering mutations during mitosis, which could result in malignant phenotype (18).

The results in the present study support the viewpoint that diagnosing epithelial dysplasia of the oral cavity is not an exact science. The present study shows the extent of interobserver agreement between the three obser-vers, when diagnosing oral epithelial dysplasia was best seen in Brothwell system with a 62% agreement (Ks= .0226), followed by WHO system with a 55.6% agreement (Ks= .0346) and least in Smith and Pindborg system with a 47% agreement (Ks= .0486). The agreement between the observers was found to be significant an all three systems (P<0.05).

Abbey et al. (19) using six American board-certified oral pathologists examined 120 oral biopsies with a range of pathologies ranging from simple hyperkeratosis to severe dysplasia. The percentage of the exact agreement among the observers ranged from 35.8% to 55.8%. However, the kappa values improved to 0.70-0.88 for inter examiner comparisons when the grading was expanded to “within one histological grade” (15).

On assessing the intraobserver variability of the three observers in each of the three systems in the present study, an overall significance was found (P<0.05). However, the ability to repeat the same diagnosis in a system was as low as 53% for observer 2 in Brothwell system and was no higher than 93.3% for observer 1 in Smith and Pindborg system.

Although the inter observer agreement between the three observers in Smith and Pindborg system was lowest, the intraobserver agreement was significant with an agreement ranging from 60.00% of observer 2 to a 93.33% agreement of observer 1. Even though the interobserver agreement between the three observers was best seen in Brothwell system, the intraobserver agreement was less significant with an agreement range from 53.33% to 66.67%. The least variability in range between the three observers was seen in this system hence validating that the interobserver variability is the least in Brothwell system.

Abbey et al. (19) in their study on intra examiner reliability in diagnosing OED found an exact agreement in 50% cases, but an agreement within one step was 90% or greater. Brothwell et al. (1) found an intraexaminer agreement varying from 84% to 94%, with kappa va-lues ranging from 0.22 to 0.78 in their study on presence/absence of dysplasia. On a five point ordinal scale their intra examiner agreement varied from 47 to 87%, with Ks ranging from 0.30 to 0.83. They explained the poor agreement in intra examiner study as a shortcoming in kappa rather than poor agreement in and of itself. Their results showed that observer bias is present which arises by the use of slightly different diagnostic thresholds and results in slightly different proportions of cases assigned to each diagnostic category.

From a statistical point of view, a discrepancy between the unweighted and weighted kappa agreement has been seen in some of the papers. Unweighted kappa statistics (Ks) tended to have lesser values than those of weighted kappa (Kw). In fact, kappa statistics measures exact agreement between two raters for nominal variables but when used with ordinal data it is influenced by the magnitude of disagreement and attributes equal importance to all disagreements. To correct this deficiency, weighted K statistics have been devised and recommended. Practically, weighted kappa resulted in a better inter and intra observer agreement values on grading oral epithelial dysplasia using five point ordinal scale grading system. A standardized statistical method in the future studies on grading dysplasia based on using the weighted kappa test would help to improve the agreement rates between observers and would better reflect the reality of the whole process (3).

The variability in diagnosis depends on which histologic and cytologic characteristics are considered to be important for the diagnosis, on the variability in the observation of these characteristics, and on the variability in the grading of these characteristics into the various categories of epithelial dysplasia. Another source of variation might be related to the difference in the understanding of these features in terms of recognition and impact on clinical outcomes (13).

The second phase of the study was carried out to categorize the individual significance of various indicators of dysplasia with respect to their reliability among various grades of dysplasia.

In the current study each indicator of dysplasia was found to be statistically significant (P<0.05) for grading dysplasia. The mean and standard deviation for each indicator of dysplasia was statistically calculated and was found to be noteworthy in giving a diagnosis of mild, moderate and severe dysplasia. Following this, the significance of each indicator of dysplasia with respect to its reliability among various grades of dysplasia was calculated. The significance of each indicator of dysplasia was found out in differentiating mild dysplasia from moderate and severe dysplasia and moderate from severe dysplasia. Drop shaped rete pegs, basal cell hyperplasia, loss of intercellular adherence and loss of polarity were not significant in differentiating moderate from severe dysplasia with a P value = 0.935, 1.000, 0.239 and 0.0999 respectively but were significant in the other two groups. Presence of bizarre mitosis was not significant in differentiating mild from moderate dysplasia (P= 0.183). Indicators in diffe-rentiating other groups were all significant.

Kujan et al. (3) in their study found out the sources of variation in grading OED lesions by reaching out to a kappa agreement on the presence/absence of each morphological characteristic of both architecture and cytology changes which was sought for by four observers divided into two studied groups (the all participating pathologists group and the oral pathologists group). The highest agreement scores were found on the following features: increased number of mitotic figures (Kappa (K) = 0.46, K= 0.53, respectively), drop shaped rete pegs (K= 0.42, K= 0.47, respectively) in the architectural changes group, whereas, increased nuclear size (K= 0.21, K= 0.34, respectively) and abnormal variation in cell shape (K= 0.20, K= 0.41, respectively) in the cytology characteristics group. On the other hand, irregular epithelial stratification and loss of polarity of basal cells from the architectural features group in addition to abnormal variation in the nuclear size, atypical mitotic figures and hyperchromatism from the cytological features group corresponded with the highest disagreement scores for both studied groups of observers.

Improvement in the standard of histopathology reporting of OED lesions can be achieved by consideration of several points. Of these paramount is a need for a universal definition of the architectural and cytological features that are the basis of any OED grading process. Also, consensus scoring process between two or more observers should be encouraged as this would not only improve the inter observer agreement but help to eliminate errors.

The underlying essence in the diagnosis of oral epithelial premalignant lesions is the concern of these lesions with regards to their malignant transformation. The empirical advantage of oral cancers being preceded by premalignant lesions is a matter of high interest for prevention of cancers. Thus, proper categorization/grading would be valuable in the overall assessment of the probability of these premalignant lesions progression towards cancer.

The present study puts forth the inherent intricacies in the grading of oral premalignant lesions. These difficulties with regards to consistency at an inter observer and an intra observer level in varying grading systems need to be further dwelled upon such that an effective unified grading protocol could be standardized to grade these dysplastic lesions.
